# Antiproliferative factor (APF) binds specifically to sites within the cytoskeleton-associated protein 4 (CKAP4) extracellular domain

**DOI:** 10.1186/s12858-017-0088-y

**Published:** 2017-09-11

**Authors:** Burzin Chavda, Jun Ling, Thomas Majernick, Sonia Lobo Planey

**Affiliations:** Department of Basic Sciences, Geisinger Commonwealth School of Medicine, 525 Pine Street, Scranton, PA 18509 USA

**Keywords:** CKAP4, APF, Surface plasmon resonance (SPR), Biomarker, Interstitial cystitis, Biosensor, Cytoskeleton

## Abstract

**Background:**

Antiproliferative factor (APF) is a sialoglycopeptide elevated in the urine of patients with interstitial cystitis—a chronic, painful bladder disease. APF inhibits the proliferation of normal bladder epithelial cells and cancer cells in vitro, presumably by binding to its cellular receptor, cytoskeleton associated-protein 4 (CKAP4); however, the biophysical interaction of APF with CKAP4 has not been characterized previously. In this study, we used surface plasmon resonance (SPR) to explore the binding kinetics of the interaction of APF and as-APF (a desialylated APF analogue with full activity) to CKAP4.

**Results:**

We immobilized non-glycosylated APF (TVPAAVVVA) to the Fc1 channel as the control and as-APF to Fc2 channel as the ligand in order to measure the binding of CKAP4 recombinant proteins encompassing only the extracellular domain (Aa 127–602) or the extracellular domain plus the transmembrane domain (Aa 106–602). Positive binding was detected to both CKAP4_126–602_ and CKAP4_106–602_, suggesting that as-APF can bind specifically to CKAP4 and that the potential binding site(s) are located within the extracellular domain. To identify the primary APF binding site(s) within the CKAP4 extracellular domain, deletion mutants were designed according to structural predictions, and the purified recombinant proteins were immobilized on a CM5 chip through amine-coupling to measure as-APF binding activity. Importantly, both CKAP4_127–360_ and CKAP4_361–524_ exhibited a fast association rate (*k*_*on*_) and a slow dissociation rate (*k*_*off*_), thus generating high binding affinity and suggesting that both regions contribute relatively equally to overall as-APF binding. Therefore, two or more as-APF binding sites may exist within the Aa 127–524 region of the CKAP4 extracellular domain.

**Conclusions:**

We determined that the CKAP4_127–360_ and CKAP4_361–524_ mutants exhibit improved binding activity to as-APF as compared to the full-length extracellular domain, making it possible to detect low concentrations of as-APF in urine, thereby establishing a foundation for a non-invasive diagnostic assay for IC. Further, these data have revealed novel APF binding site(s) suggesting that targeting this region of CKAP4 to inhibit APF binding may be a useful strategy for treating IC-related bladder pathology.

## Background

Antiproliferative factor (APF) is a lipophilic, nine-residue sialoglycopeptide with an amino acid sequence identical to the sixth transmembrane (TM) domain of Frizzled-8, a Wnt ligand receptor. APF is present in the urine of patients with interstitial cystitis (IC)—a chronic, painful bladder disease with a poorly understood etiology [1, 2]. Exposing cells to APF in vitro dramatically alters gene expression and inhibits proliferation of normal bladder epithelial cells and cancer cell lines including bladder (T24) and cervical (HeLa) adenocarcinoma, mimicking critical aspects of the pathology of the bladder epithelium in IC patients.

APF’s potency to inhibit proliferation (IC_50_ ~ 1 nM), ability to alter gene expression, and the requirement for a hexosamine-galactose disaccharide linked in a specific α configuration to the backbone peptide for activity suggest that its cellular effects are mediated by binding to a TM receptor [3]. Indeed, Conrads et al. (2006) identified cytoskeleton-associated protein 4 (CKAP4) as a cellular receptor for APF in bladder epithelial cells using a pull-down assay approach and showed co-localization of CKAP4 and rhodamine-labeled synthetic APF in both cell membrane and perinuclear areas by immunofluorescent confocal microscopy. CKAP4 (also known as p63, CLIMP-63, and ERGIC-63) is a 63 kDa, nonglycosylated, reversibly palmitoylated, type II TM protein. Original studies described it as an endoplasmic reticulum (ER) resident protein that anchors the rough ER to microtubules in epithelial cells [4, 5] and plays an essential role in the maintenance of ER structure [6–8]. Reducing CKAP4 expression with either siRNA or anti-CKAP4 antibodies in competition experiments or inhibition of CKAP4 palmitoylation reverses APF’s effects on cell proliferation and gene expression [3, 9], suggesting that CKAP4 functions as a cell membrane receptor for APF. More recently, it was shown that changes in MMP2, p53 and CCN2 protein expression and Akt/GSK3β/β-catenin phosphorylation in response to APF treatment were all specifically abrogated following CKAP4 siRNA knockdown in T24 bladder carcinoma cells [10, 11]. These data indicate that CKAP4 is essential for mediating the signaling and antiproliferative activity of APF.

Interestingly, CKAP4 has also been identified as a cell membrane receptor for three additional, seemingly unrelated ligands—tissue plasminogen activator (tPA), surfactant protein A (SP-A), and Dickkopf1 (DKK1). Razzaq et al. (2003) isolated CKAP4 from ^125^I–labeled vascular smooth muscle cell (VSMC) surface proteins using an affinity chromatography procedure based on tPA binding characteristics. Evidence supporting CKAP4’s functionality as the tPA binding protein was established when a monoclonal antibody against CKAP4 blocked tPA from binding to VSMC [12]. Additionally, COS cells transfected with an N-terminally truncated CKAP4 mutant constitutively localized to the plasma membrane resulted in a > 7 fold increase in plasminogen activity that was reversed with an anti-CKAP4 antibody [5, 12]. Gupta et al. (2006) later provided evidence signifying CKAP4’s role as a plasma membrane receptor for SP-A using two chemical cross linkers and identification by mass spectrometry and Western blotting. The interaction between CKAP4 and SP-A was confirmed by co-immunoprecipitation experiments and the use of an antibody against CKAP4 which obstructed SP-A’s ability to block surfactant secretion [13]. SP-A characteristically binds to alveolar type II cells via calcium dependent (specific) binding and calcium independent (nonspecific) binding. Incubation with an antibody against CKAP4 blocked SP-A’s specific binding, but did not alter the nonspecific interactions. Similar results were obtained when CKAP4 antibody was used in experiments that exposed type II cells to cAMP, which increased SP-A binding. Additionally, siRNA knockdown of CKAP4 resulted in an inhibition of SP-A specific binding and further demonstrated CKAP4’s ability to bind to SP-A [14]. More recently, Kimura and colleagues identified CKAP4 as a receptor for DKK1 (Dickkopf1), a secreted protein that antagonizes Wnt signaling [15]. They showed that DKK1 signaling through CKAP4 promotes cellular proliferation through activation of the PI3K/AKT pathway. Evidence sup porting CKAP4 functioning as a DKK1 receptor include its localization to the apical membrane of polarized MDCK cells, its internalization by DKK1 through a clathrin-mediated pathway, and binding of DKK1 to the CKAP4 extracellular domain [16].

The effects that APF has on cells are remarkable and unique among the reported ligands for CKAP4. Foremost, APF profoundly inhibits the growth of normal bladder epithelial cells, bladder carcinoma cells, and HeLa cells in vitro with an IC_50_ of ~1 nM. APF is the only CKAP4 ligand known to cause its internalization, nuclear translocation [17, 18], and subsequent changes in the transcription of at least 14 genes that are involved in regulating proliferation and tumorigenesis (including E-cadherin, cyclin D1, vimentin, α-1-catenin, HB-EGF, α-2-integrin, p53, occludin, ZO-1, MMP2, β-catenin, COX-2, CCN2). Changes in the expression of these and other potential genes underlie the APF-induced transition to a non-proliferative, more differentiated phenotype as is observed in explanted bladder epithelial cells from IC patients. However, the mechanism(s) by which CKAP4 mediates APF’s cellular effects is unknown, and characterization of their direct interaction has not been examined previously.

In this study, we sought to explore the binding kinetics of the interaction of APF and as-APF (a desialylated APF analogue with full activity) to CKAP4 using surface plasmon resonance (SPR) technology. SPR has been used to quantify many types of molecular interactions, including recombinant proteins and peptides [19]. To determine whether CKAP4 could interact specifically with APF/as-APF at high affinity, we designed a series of recombinant CKAP4 proteins encompassing the extracellular domain and regions within the extracellular domain. These proteins were immobilized on a CM5 chip surface through amine coupling and the interaction with APF/as-APF was measured. Both APF peptides bound to CKAP4 with high specificity compared to control APF peptide; however, we observed much stronger binding of CKAP4 to as-APF compared to APF. Importantly, we determined that the CKAP4 extracellular domain is required for as-APF binding, and that the primary APF binding site is located within amino acids 127–524 of this region. The binding of as-APF to the CKAP4 extracellular domain was further supported by mass spectrometry data. Thus, these data have revealed novel APF binding site(s), suggesting that targeting this region of CKAP4 to inhibit APF binding may be a useful strategy for treating IC related bladder pathology.

## Methods

### Materials

Anti-His antibody (Cat# 200–301-382) was purchased from Rockland (Gilbertsville, PA). Anti-CKAP4 G1/296 (Cat# ALX-804-604) was from Enzo Life Sciences (Farmingdale, NY). Anti-CKAP4 corresponding to internal sequence amino acids 216–265 (ab84712) was from Abcam (Cambridge, MA). Anti-CKAP4 corresponding to internal sequence amino acids 375–425 (Cat# A302-256A), 500–550 (Cat# A302-257A), and 552–602 (Cat# A302-258A) were from Bethyl Laboratories (Montgomery, TX). APF peptide (Neu5Acα2 − 3Galβ1 − 3GalNAcα-O-TVPAAVVVA) at 93% purity was purchased from New England Peptides (Gardner, MA), and as-APF (asialo APF, Galβ1 − 3GalNAcα-O-TVPAAVVVA) at 88.5% purity was synthesized by Peptides International (Louisville, KY). Control APF peptide (TVPAAVVVA) at 95% purity was synthesized by GenScript.

### Generation of CKAP4 recombinant proteins

CKAP4 deletion mutant constructs consisting of amino acids 106–602, 127–360, 361–524, and 525–602 regions were generated by PCR and subcloned into the pET-15b bacterial expression vector (Novagen). The 127–602 mutant was subcloned into the pRSET-B vector (Life Technologies). All of the mutants were used to transform BL21(DE3)pLysS chemically competent *E. coli* (Thermo Fisher). Transformed *E. coli* cells were plated on to LB agar plates containing 100 μg/ml ampicillin and 34 μg/ml chloramphenicol. Successful transformants were expanded in LB/Amp (100 μg/ml ampicillin) medium containing 34 μg/ml chloramphenicol and used to make glycerol stocks, which were kept at −80 °C until needed. Saturated cultures were prepared by inoculating LB/Amp medium (containing chloramphenicol) with glycerol stocks, and grown overnight at 37 °C.

For expression, overnight cultures were diluted 1:20 in LB/Amp and grown at 37 °C with shaking at 250 rpm until they reached the exponential growth phase (OD_600 nm_ of ~0.4). Protein expression was induced by adding IPTG (1 mM final concentration) and growth was continued for an additional 3 h at 37 °C. All subsequent steps were carried out at 4 °C. Cells were harvested by centrifugation. Lysates were prepared by resuspending the cell pellets in PBS buffer containing 20 mM imidazole and EDTA-free protease inhibitor cocktail (Thermo Fisher), followed by sonication (on ice) until lysates appeared clearer. The lysates were clarified by centrifugation at 10,000 x g for 15 min, and supernatants were subsequently used for protein purifications. Histidine-tagged CKAP4 deletion mutants were purified by immobilized metal affinity chromatography (IMAC) using TALON Superflow chromatography medium (GE Healthcare) by standard batch-purification procedures. Eluates containing target proteins (as confirmed by SDS-PAGE and Western blotting) were pooled, and then buffer exchanged into PBS using Amicon Ultra centrifugal filter units (Millipore) to yield the final preparations. CHAPS detergent (10 mM) was used in all buffers during lysate preparation, purification, and final preparation for the CKAP4_106–602_ mutant.

FL-CKAP4 was expressed in HEK 293 T cells (ATCC) and similarly purified by IMAC. Briefly, HEK 293 T cells were transfected with pcDNA3.1/V5-His TOPO vector (Life Technologies) expressing FL-CKAP4 using X-tremeGENE 9 transfection reagent (Roche) according to manufacturer’s protocol. Cells were harvested after transient expression (24 h) and held at −80 °C before proceeding to purification. Cell lysate was prepared in PBS buffer containing 10 mM CHAPS, 20 mM imidazole and EDTA-free protease inhibitor cocktail and sonicated before proceeding to protein purification, as described above. All purified proteins were quantitated by the BCA method according to the manufacturer’s protocol using BSA as the standard (ThermoScientific). Because of the varied amino acid compositions of the purified recombinant proteins, the UV (λ280nm) absorbance method was also used for reference.

### Gel electrophoresis and western blotting

Proteins were separated by electrophoresis using 10% or 4–12% Bis-Tris gels (GenScript) and subsequently stained using Pierce Silver Stain Kit (Thermo Fisher) or used for Western blotting. Protein transfer to nitrocellulose membranes was done using standard procedures. Membranes were blocked with TBST buffer (Tris-buffered saline, pH 7.4, with 0.05% Tween-20 containing 5% (*w*/*v*) non-fat dry milk) for 1 h at room temperature; primary antibody was incubated overnight at 4 °C and secondary antibody for 1 h at room temperature. Membranes were developed with SuperSignal West Pico chemiluminescent substrate (Thermo Fisher) and visualized by autoradiography.

### Surface plasmon resonance assays

All SPR experiments were performed on a Biacore X-100 instrument (GE Healthcare) with the chip surface at 25 °C using PBS containing 0.01% Tween-20 as running buffer. CKAP4 recombinant proteins were immobilized individually either by Ni^2+^/nitrilotriacetic acid (NTA) chelation on Sensor Chip NTA or by amine-coupling on Sensor Chip CM5, on the Fc2 channel. For amine-coupling, the sensor surface on both flow cells was activated for 7 min with a 1:1 mixture of 0.1 M EDC (1-ethyl-3-(3-dimethylaminopropyl) carbodiimide hydrochloride) and 0.1 M NHS (N-hydroxysuccinimide) at a flow rate of 5 μl/min. Immobilization of CKAP4 proteins (on Fc2) was performed in 10 mM sodium acetate buffer, at either pH 4.5, 5.0, or 5.5 based on preliminary pH scouting runs, for optimal binding. Unreacted NHS groups were quenched with a 7 min injection of 1 M ethanolamine pH 8.5. The Fc1 channel was treated similarly but without CKAP4 and served as a reference. Upon stabilization of the baseline signal, multi-cycle kinetics assays were performed with injections of as-APF as analyte at various concentrations (2.5–40 μM) in running buffer at 30 μl/min for 2 min, immediately followed by a 3 min dissociation phase. Manual runs with injections of as-APF, APF, or APF control peptide (non-glycosylated) as flow analytes were also performed at 30 μl/min for varied amounts of time. Between analyte injections, the sensor surface was regenerated with short (30 s) pulses of 0.2% (*v*/v) SDS and 2.5 M NaCl. The data were analyzed using Biacore Evaluation software V2.0. Kinetic constants were estimated by employing various fitting models, including heterogeneous ligand binding, 1:1 binding, and two-state binding.

For experiments using spiked urine, CKAP4_127–360_ was immobilized on the Fc2 channel of a CM5 chip by amine-coupling, as the ligand. The Fc1 channel was similarly treated but without CKAP4, to serve as reference. A control urine sample from a healthy donor was mixed 1:1 with as-APF stock (200 μM) solution. Various dilutions of this mixture in SPR running buffer were prepared to yield desired as-APF final concentrations, and then injected over the sensor chip surface. Corresponding dilutions of unspiked control urine were run under identical conditions to account for background interference from urinary constituents. To ensure identical running conditions, all dilutions were adjusted to 1X SPR running buffer from a 10X stock. Sensorgrams were corrected for background interference from urinary constituents by subtracting the RU traces resulting from unspiked urine injections from the RU traces resulting from the corresponding urine dilutions with spiked as-APF, and subsequently replotted after subtracting the RU trace from the reference (Fc1) channel.

### Detection of APF-CKAP4 interaction by LC-MS

Equal amounts of purified HT-CKAP4 recombinant proteins were immobilized to 20 μl bed of TALON Superflow resin. Reactions without protein served as controls. The resin was then washed/equilibrated three times with PBS. CKAP4-immobilized resin was then incubated with 100 μl of either APF or as-APF (at 40 μM final concentration) in PBS for 90 min at room temperature with rotation. The resin was then washed five times with PBS. Bound proteins were eluted in 50 μl PBS containing 175 mM imidazole. Eluates were filtered through standard polyethylene filter to remove any resin particles. Samples were acidified to pH 3 with 10% formic acid, and 10 μl of samples were desalted using stage tip packed with Empore C18 (3 M) and vacuum dried. After re-solubilization in 10 μl 0.1% TFA, 5 μl was analyzed by nano LC-MS/MS with a Dionex RSLC on Q Exactive™ HF (Thermo Fisher). Samples were loaded onto a 100 μm × 2 cm trap packed with Magic C18AQ, 5 μm 200 A (Michrom Bioresources Inc., Auburn, CA) and washed with Buffer A (0.2% formic acid) for 5 min with flow rate of 10 μl/min. The trap was brought in-line with the analytical column (Magic C18AQ, 3 μm 200 A, 75 μm × 50 cm) and peptides were fractionated at 300 nL/min flow rate with a linear gradient of 4–50% Buffer B (0.16% formic acid, 80% acetonitrile) in 25 min. Mass spectrometry data was acquired using a data-dependent acquisition procedure with a cyclic series of a full scan.

### Cell proliferation assay

T24 bladder carcinoma cells (ATCC #HTB-4; Manassas, VA) were maintained in HyClone-formulated McCoy’s 5A modified media (Catalog #SH30525.01) supplemented with 10% fetal bovine serum (FBS) (Atlanta Biologicals, Flowery Branch, GA), 100 U/ml penicillin, and 100 μg/ml streptomycin (Invitrogen, Carlsbad, CA). Cell proliferation was measured using the XTT Cell Proliferation Assay Kit (ATCC). T24 cells were seeded at 1 × 10^3^–4 × 10^3^ per well in a 48-well plate in 300 μl/well McCoy’s supplemented medium. Cells were serum-starved for 24 h and then treated with either 20 nM APF, 20 nM as-APF, or 25 μM H_2_O_2_ (antiproliferative control) diluted in serum-free McCoy’s medium. Cells were incubated at 37 °C in a 5% CO_2_ atmosphere for an additional 96 h, after which 150 μl of activated–XTT solution was added to each well followed by an additional 3 h incubation. Absorbance was then measured at 475 nm and 660 nm using an M5 microplate reader (Molecular Devices, Sunnyvale, CA), and the specific absorbance was calculated. *P* values were assigned using unpaired t-test with Welch’s correction. The assay was repeated four independent times.

## Results

### as-APF binds specifically to the CKAP4 extracellular domain

CKAP4 is a single TM domain protein that has been identified as a high-affinity, cell-surface receptor for APF [3]. While studies to date have demonstrated that CKAP4 expression and palmitoylation are required for APF activity in vitro, characterization of their putative, direct interaction has not been examined previously. To determine whether CKAP4 could interact specifically and with high affinity to APF or as-APF (a desialylated APF analogue with full activity), we expressed and purified full-length, His-tagged (HT) CKAP4 in mammalian cells and measured binding activity by SPR. However, we did not detect appreciable binding activity between full-length CKAP4 and APF or as-APF. We speculated that this might be due to protein misfolding or interference of the cytoplasmic and TM domains with the correct folding of the extracellular domain of CKAP4.

**Fig. 1:**
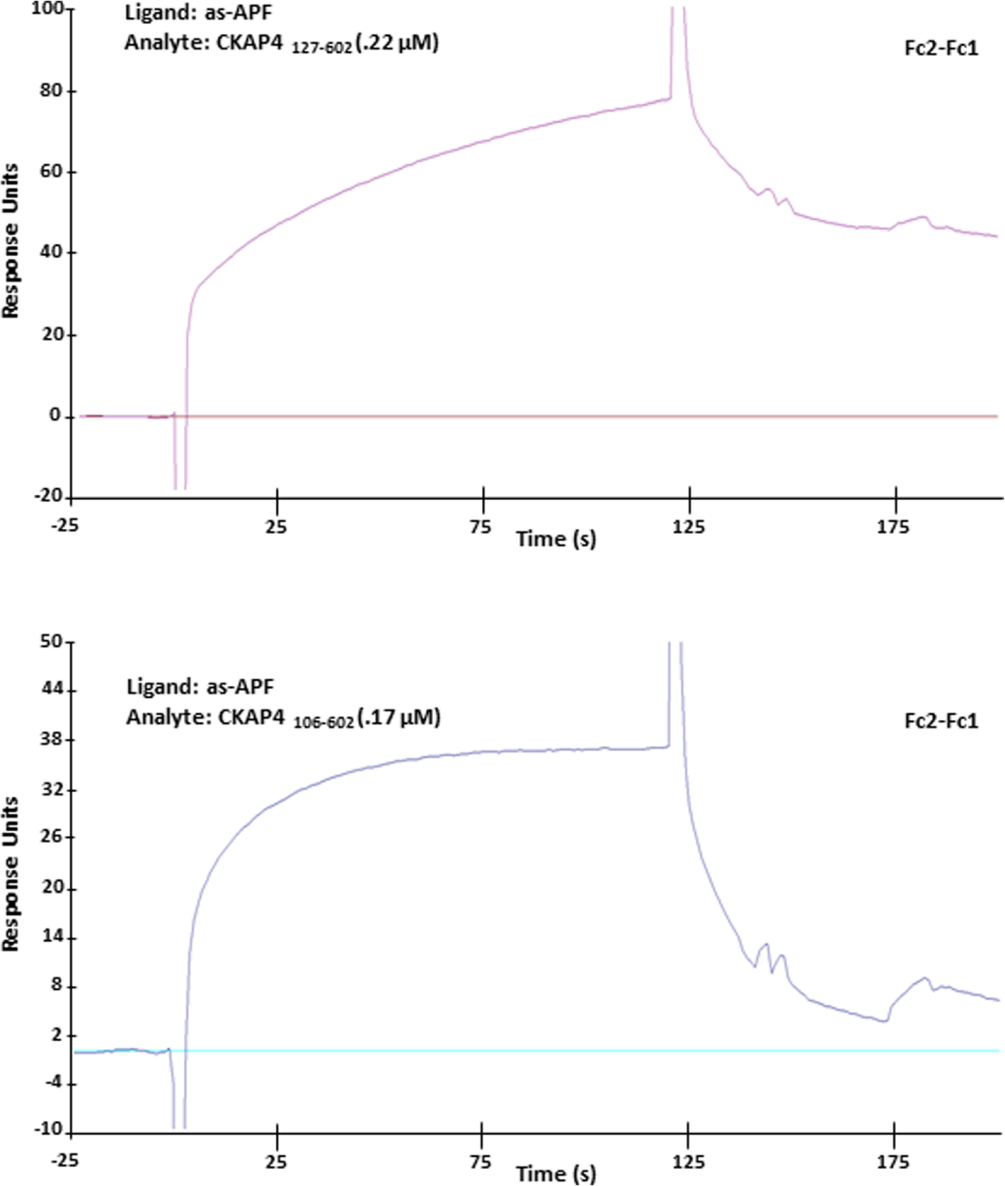
as-APF binds directly and specifically to the CKAP4 extracellular domain. Non-glycosylated APF control peptide (TVPAAVVVA) was immobilized to the Fc1 channel of a CM5 chip through amine-coupling as the control (immobilized RU = 530); as-APF was immobilized to the Fc2 channel as the ligand (immobilized RU = 347). Purified CKAP4 extracellular domain CKAP4_127–602_ (0.22 uM) and the extracellular domain with the transmembrane domain CKAP4_106–602_ (0.17 uM) were used as the analytes for the binding assay by SPR. Adjusted sensorgrams (Fc2-Fc1) are shown above

CKAP4 has a 106-amino acid long cytosolic tail, a single, 21-amino acid TM domain, and a large extracellular domain of 474 amino acids. Since TM proteins that function as receptors traditionally have ligand binding site(s) located within their extracellular domain, we engineered a recombinant CKAP4 protein encompassing only the extracellular domain (Aa 127–602) tagged at the C-terminus with a His-tag (CKAP4_127–602_) and the extracellular domain plus the TM domain (CKAP4_106–602_) with N-terminal His-tag to measure binding to APF/as-APF. When both mutants were used as the ligand immobilized on a NTA (nitrilotriacetic acid) sensor chip surface, they did not bind robustly to APF or as-APF. To rule out the possibility that the fixed orientation of CKAP4 on the NTA chip due to immobilization through the His-tag could account for the lack of binding, we used an alternate strategy immobilizing APF or as-APF to a CM5 chip as the ligand to test their binding to the CKAP4 ED mutants. pH scouting revealed that APF had limited/insufficient immobilization to the CM5 chip, whereas as-APF and control APF (non-glycosylated APF without biological activity) were efficiently immobilized (data not shown). This disparity is likely due to the unique structure of APF, which possesses only one –NH2 group at its N-terminus that can be amine-coupled to the CM5 chip and a Neu5Ac group that may reduce accessibility of –NH2. As a result, we immobilized non-glycosylated APF (TVPAAVVVA) to the Fc1 channel as the control and as-APF to Fc2 channel as the ligand in order to measure the binding of the CKAP4 extracellular domain mutants. As shown in the adjusted sensorgram (Fc2-Fc1) in Fig. 1, positive and specific binding was detected to both CKAP4_126–602_ and CKAP4_106–602_, suggesting that as-APF can bind directly to CKAP4 and that the potential binding site(s) are located within the extracellular domain. Different binding kinetics were observed for each extracellular domain mutant, as CKAP4_127–602_ showed a faster association rate than CKAP4_106–602_, suggesting that the TM domain might interfere with the conformation of the extracellular domain.

**Fig. 2:**
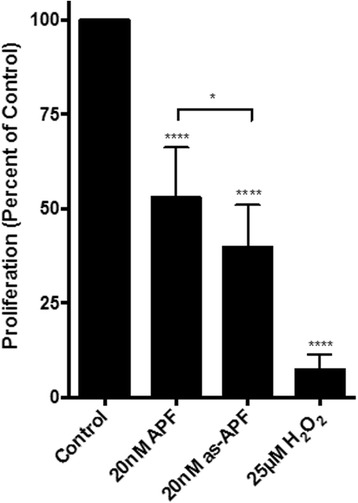
Verification of antiproliferative activity of APF and as-APF by a cell-based assay. The biological activity of the as-APF and APF peptides was examined by measuring T24 bladder carcinoma cell proliferation using the XTT Cell Proliferation Assay. Metabolically active cells reduce XTT to its formazan derivative, causing a colorimetric change in the medium that can be measured by a spectrophotometer. The relative activities of APF and as-APF are shown and normalized to the control. We observed that both APF and as-APF are biologically active and significantly inhibit cell proliferation (*P* < 0.0001). The proliferation of T24 cells decreased by 60.16 ± 10.67% after treatment with 20 nM as-APF, while APF (20 nM) reduced proliferation by 47.08 ± 12.88%. The difference in the antiproliferative effect of as-APF and APF was determined to be significant (*P* = 0.0103). *P* values were assigned using unpaired t tests with Welch’s correction. The proliferation assay was repeated at least four independent times

To confirm the biological activity of the as-APF and APF peptides, we measured their effect on T24 bladder carcinoma cell proliferation. Cells were treated with 20 nM APF or as-APF for 96 h and the rate of cell growth was measured using the XTT proliferation assay. As shown in Fig. 2, both APF and as-APF are biologically active and significantly inhibit cell proliferation (*P* < 0.0001). The rate of growth of T24 cells was reduced by 47% with APF and by 60% with as-APF, relative to cells treated with vehicle alone; moreover, the difference in the antiproliferative effect of as-APF and APF was determined to be significant (*P* = 0.0103), indicating that as-APF had a more potent biological effect on cellular proliferation. Other studies, albeit not in complete agreement with ours, have reported that the antiproliferative effect of as-APF and APF are more or less equipotent [2]. Thus, while the carbohydrate moiety of APF is an absolute requirement for its biological activity as noted earlier, the activity does not seem to be dependent on the terminal sialic acid. In light of such findings, we focused on as-APF to further investigate its binding characteristics with CKAP4.

### Generation of CKAP4-ED deletion mutants to define the region of APF binding

**Fig. 3:**
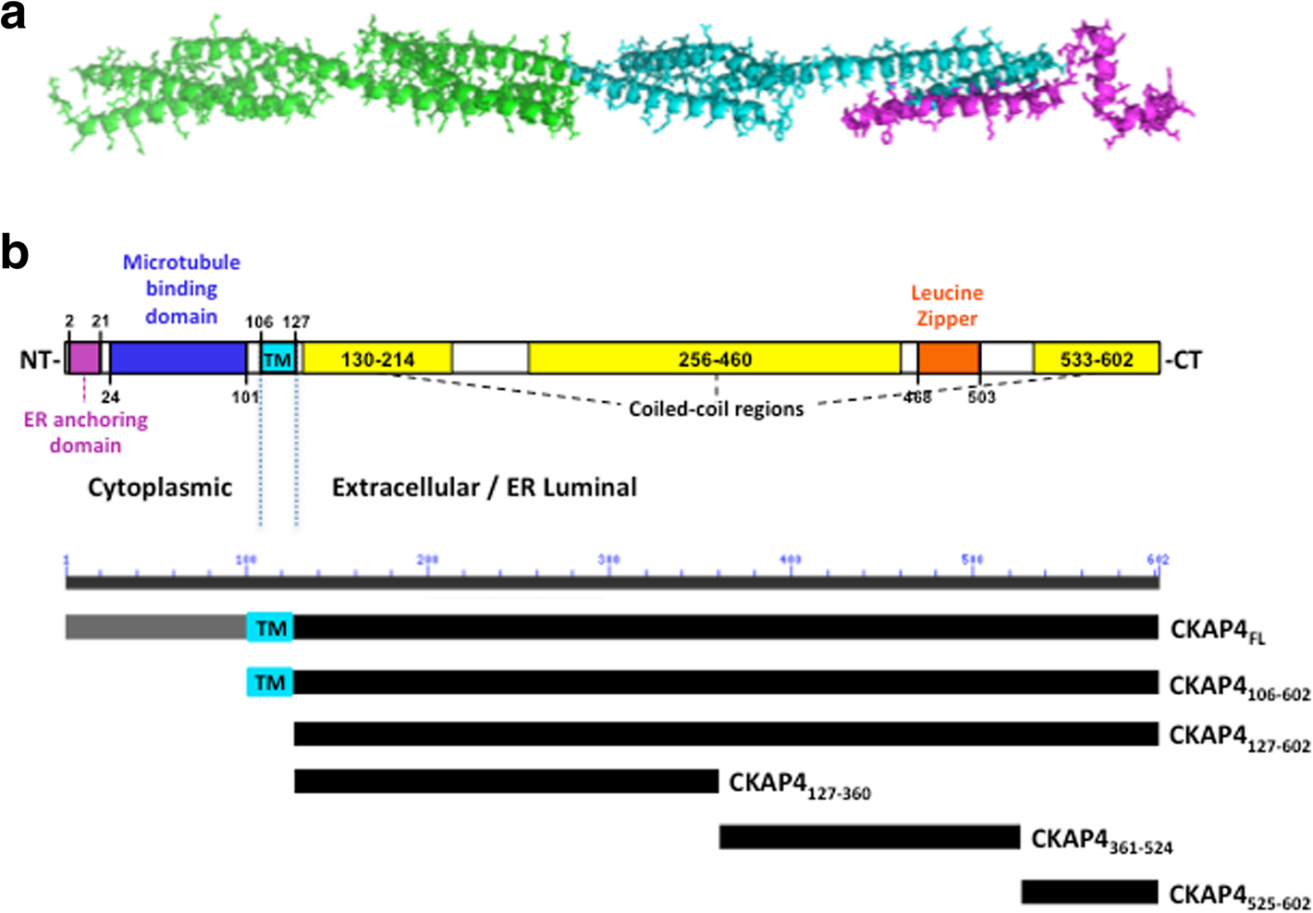
Predicted CKAP4 secondary structure and the deletion mutants used in this study. **a** Secondary structure of the CKAP4 extracellular domain (Aa 127–602) as predicted by the I-Tasser program in the protein model portal (PMP) [20, 21]. **b** Schematic diagram of full-length CKAP4 indicating structural characteristics of the protein and the corresponding schematics of the CKAP4 deletion mutants. The N-terminal domain consists of the cytoplasmic portion (Aa 1–105) and the C-terminal domain consists of the extracellular/ER luminal portion (Aa 106–602) that includes the TM domain (Aa 106–127) and contains multiple, coiled-coil domains

**Fig. 4:**
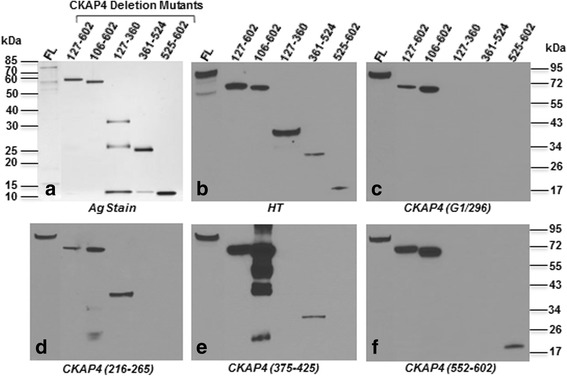
Characterization of purity and antigenicity of CKAP4 constructs. **a** SDS-PAGE of normalized amounts of purified CKAP4 constructs followed by silver staining. **b–f** Western blots using anti-His tag, anti-CKAP4 (G1/296), anti-CKAP4 (epitope region: 216–265), anti-CKAP4 (epitope region: 375–425), and anti-CKAP4 (epitope region: 552–602) primary antibody, respectively. Separate blots were used for each antibody. Molecular weight standards are indicated on left and right

To identify the primary APF binding site(s) within the CKAP4 extracellular domain, we used a deletion mutant approach to narrow down the region of binding. Using structural prediction software [Protein Model Portal [20] and I-Tasser [21]], we analyzed the CKAP4 extracellular domain and determined the optimal strategy for generating mutants of this region while preserving structural and functional domains. As shown in Fig. 3a, four relatively rigid, α-helix bundles or units span the extracellular domain of CKAP4; the region closest to the C-terminus of the extracellular domain has a more random/loose structure. Based on this predicted secondary structure, we divided the extracellular domain into three fragments as shown in Fig. 3b. Each amino acid fragment—127–360, 361–524, and 525–602—was subcloned into a pET-15b bacterial expression vector through NdeI (5′) and XhoI (3′) restriction sites and subsequently expressed in BL21 (pLysS) bacteria. These three CKAP4-ED deletion mutants (CKAP4_127–360_, CKAP4_361–524_, CKAP4_525–602_) along with CKAP4-ED (CKAP4_127–602_) and the CKAP4-ED variant containing the TM domain (CKAP4_106–602_) were purified by histidine-tag based affinity chromatography, dialyzed, and assessed qualitatively by SDS-PAGE and Western blot analysis. As shown in Fig. 4 (a and b), all of the CKAP4 recombinant, mutant proteins were of sufficient purity and the correct predicted size. To further confirm the purity and antigenicity of these proteins, CKAP4 antibodies directed against different regions of CKAP4-ED were used for antibody mapping. All of the CKAP4 mutants generated corresponding reactivity toward their epitope regions (Fig. 4c–f). The G1/296 monoclonal antibody, whose epitope is unknown, only showed reactivity toward CKAP4 proteins with an intact extracellular domain. These data demonstrate that the purified mutant proteins are of high quality and ensure the reliability of identification of primary APF binding site(s) in this study.

### The primary APF binding site(s) are located within the Aa 127–524 region of CKAP4

To assess the as-APF binding activity of the purified CKAP4 deletion mutants, each protein was separately immobilized on a CM5 sensor chip through amine coupling, and SPR responses against APF control peptide and various as-APF concentrations were observed. We used this reverse approach to assess as-APF binding kinetics because the concentration of as-APF synthetic peptide could be calculated more accurately than the purified CKAP4 deletion mutants, which vary in amino acid composition. Further, using CKAP4 as the biosensing ligand suits our long-term goal to develop a SPR assay to measure APF/as-APF in biological samples.

**Fig. 5:**
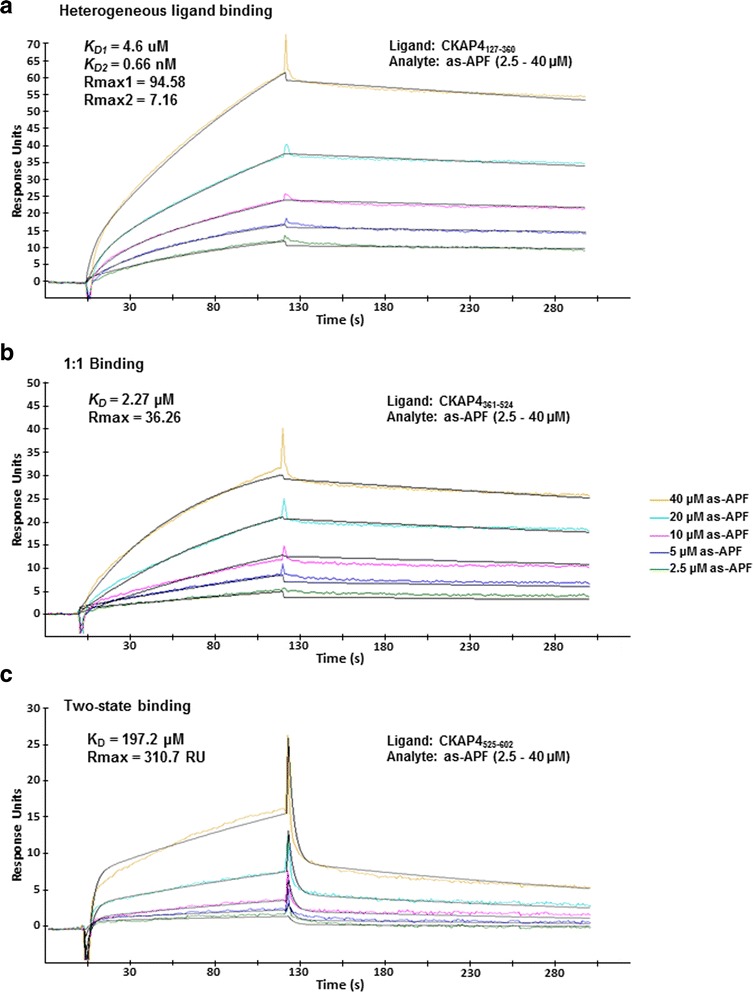
Binding kinetics of as-APF interaction with CKAP4 extracellular domain deletion mutants. Three CKAP4 ED deletion mutants were immobilized onto the Fc2 channel of separate CM5 chips, using amine-coupling, as the ligands; the Fc1 channels were treated similarly but without the protein, serving as reference. Prior to the kinetics assay, binding of each mutant to the APF control peptide was tested, and no specific binding was detected. Multi-cycle kinetics assays were then performed by the injection of various concentrations of as-APF (2.5–40 μM, colored lines) over the CM5 chip surfaces on which CKAP4_127–360_ (**a**), CKAP4_361–524_ (**b**), and CKAP4_525–602_ (**c**) were immobilized via amine-coupling. The adjusted sensorgrams (Fc2-Fc1) were overlaid to calculate binding kinetics. The data were fit using Biacore Evaluation software, and the fitting curves (black lines) as well as the parameters of binding kinetics are shown where applicable. The immobilized RU for each ligand is: CKAP4_127–360_ at 352 RU, CKAP4_361–524_ at 585 RU**,** and CKAP4_525–602_ at 1914 RU. The heterogeneous ligand model is the best fit for 5A (Chi^2^ = 0.893); the 1:1 binding model is the best fit for 5B (Chi^2^ = 0.874); and the two-state binding model is the best fit for 5C (Chi^2^ = 0.446)

As shown in Fig. 5, the CKAP4_127–360_, CKAP4_361–524_, and CKAP4_525–602_ mutants exhibited specific binding to as-APF. Mutants 127–360 and 361–524 possessed higher binding affinity over mutant 525–602. The binding data for each mutant were fit using various models to achieve the best statistical results for binding to as-APF, and the detailed kinetics information was included in each panel (Fig. 5a–c). The interaction of as-APF with CKAP4_127–360_ presented as heterogeneous ligand binding (Fig. 5a), suggesting that CKAP4_127–360_ may present in more than one conformational state to bind as-APF; thus, there might be multiple binding sites in this region. According to the principle of binding response described by the eq. (1): R_max_ = (MW_A_/MW_L_)* R_L_*Sm (where Rmax is the maximum binding, RL is the immobilized RU of ligand, MWA and MWL are the molecular weights of the analyte and ligand respectively, and Sm is the binding stoichiometry), the calculated Sm for CKAP4_127–360_ and as-APF binding is 7.1, suggesting there are multiple as-APF binding sites in this region. The precise number of binding sites will be determined in a future project by the isothermal titration calorimetry (ITC) method, where both analyte and ligand are in the free solution state without immobilization, thus the stoichiometry can be measured more accurately. Based on the remarkable binding affinity increase from K_D1_ to K_D2_, there may be allosteric or synergistic effects involved in the CKAP4_127–360_ and as-APF interaction. On the other hand, the interaction between CKAP4_361–524_ and as-APF showed a 1:1 binding model (Fig. 5b), implying that this region may have a relatively rigid structure and more uniform conformation. The calculated Sm based on the eq. (1) above is also 1, suggesting there might be only 1 binding site in this region. Despite some differences in the binding models, both CKAP4_127–360_ and CKAP4_361–524_ exhibited similar binding kinetics, showing fast association rates (*k*_*on*_) and a slow dissociation rates (*k*_*off*_), therefore generating high binding affinities (*K*_*D*_ = *k*_*off*_ /*k*_*on*_; for CKAP4 _127–360_, K_D1_ = 4.61 μM, K_D2_ = 0.66 nM; and K_D_ for CKAP4 _361–524_ = 2.3 μM; Fig. 5a and b, respectively). These binding affinities are within the range of what would be considered a strong intermolecular interaction, suggesting that the primary APF binding site(s) are located within the Aa 127–524 region of the CKAP4 extracellular domain. In contrast, the interaction of as-APF with the CKAP4_525–602_ ligand (Fig. 5c) displayed different binding kinetics with a slower association phase and a rapid dissociation phase. The best fitting model for this interaction was two-state binding, yielding a K_D_ of 197 μM and a much lower affinity. The two-state binding also indicates the possibility of conformational change in CKAP4_525–602_ upon binding to as-APF, which was partially supported by the calculated Sm of 1.5 for the CKAP4_525–602_ and as-APF interaction. Thus, this region of CKAP4 may represent a flexible conformation to coordinate the overall conformational change during the binding of intact protein to as-APF. Taken together, we determined that the CKAP4_127–360_ and CKAP4_361–524_ mutants exhibit normal binding to as-APF with proportional response to increasing doses of analyte, suggesting that these two regions contribute relatively equally to overall APF binding, and that multiple primary binding sites may exist within CKAP4. In contrast, the binding response of CKAP4_525–602_ is not proportional to concentration changes, especially below 10 uM, suggesting the complexity of binding in this region. Given its inherent difficulty as a membrane protein, the complete delineation of APF binding sites within the CKAP4 extracellular domain will require further investigation, possibly by orthogonal methods.

**Fig. 6:**
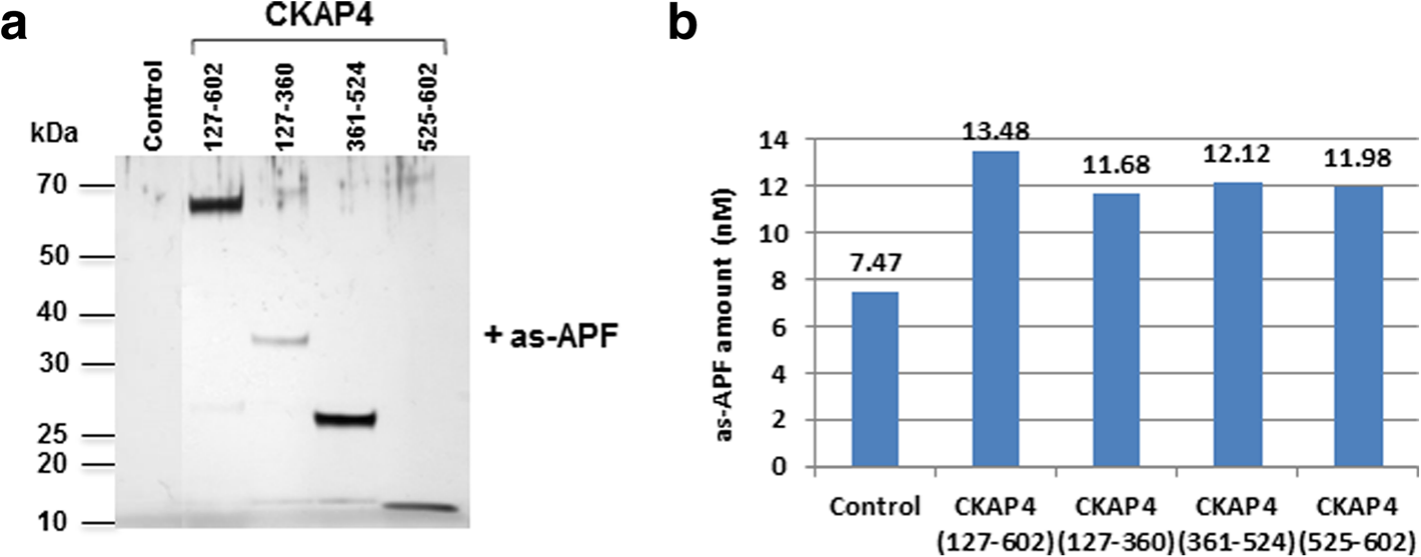
Detection of as-APF binding to CKAP4 mutants by LC-MS. Various CKAP4 mutant-immobilized His-tag affinity beads were used to interact with as-APF, and the captured as-APF was analyzed by MS as described in “Materials and Methods”. **a** The protein staining of SDS-PAGE to show the efficiency of experiment; **b** The quantitation of as-APF bound to various CKAP4 mutants based on the as-APF standard curve (not shown) also performed with the same LC-MS conditions

To corroborate the results of the SPR-based assay, we also employed a ‘bead-based’ immobilization approach in conjunction with mass spectrometry (Fig. 6). Briefly, CKAP4 mutant proteins were each immobilized as bait on His-tagged affinity beads and incubated with as-APF. After washing, the CKAP4-as-APF complex was eluted from the beads, and the potentially bound as-APF was quantified by LC-MS (Fig. 6a and b). LC-MS quantitation indicated that all four mutants showed a higher amount of bound as-APF than the APF control peptide, thus supporting the findings by SPR. It should be noted that this approach was undertaken only as a qualitative confirmation as it does not represent any kinetic parameters of the interactions.

### Binding of as-APF to the CKAP4 extracellular domain is specific

**Fig. 7:**
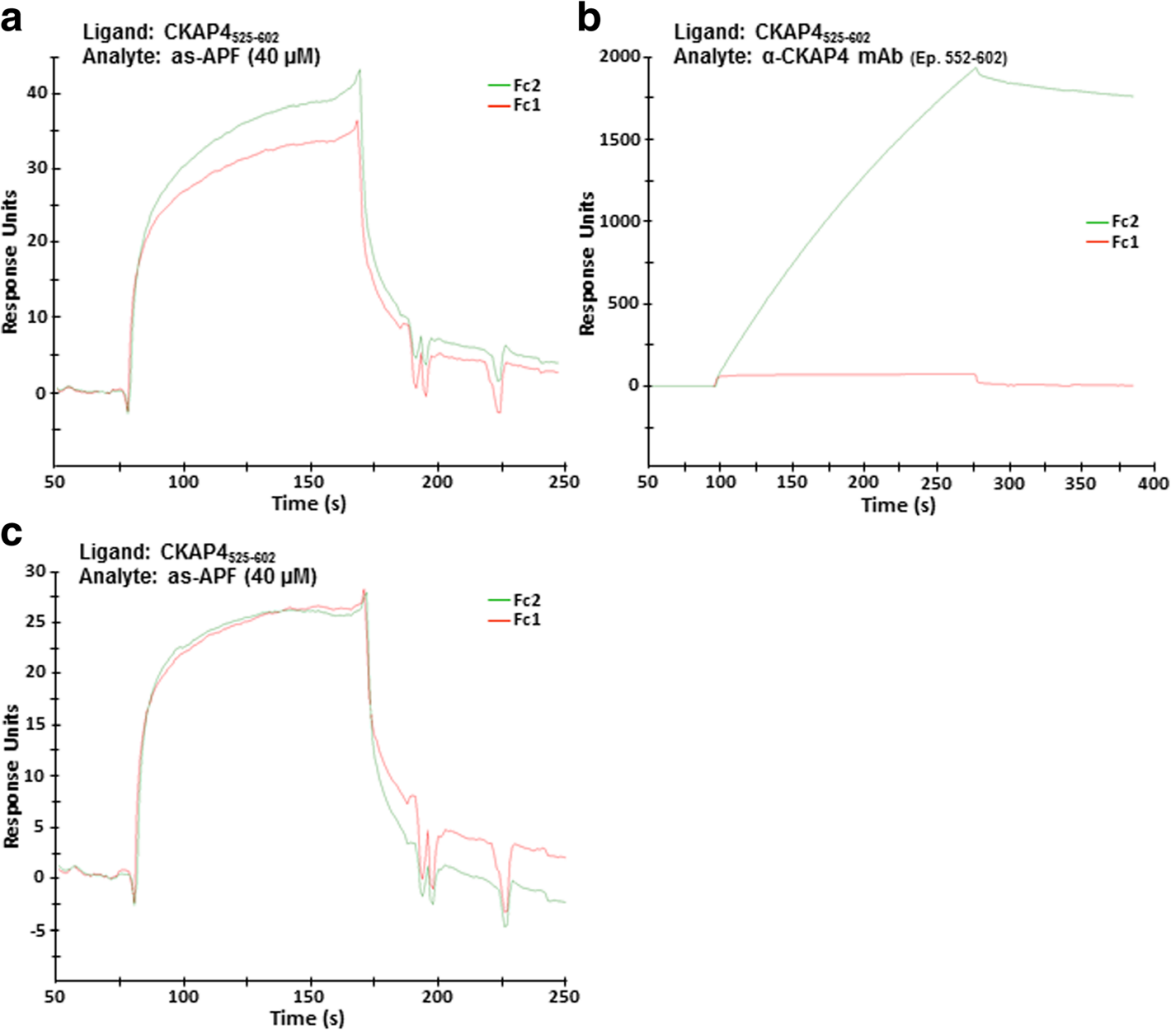
Binding of as-APF to CKAP4_525–602_ mutant is blocked by epitope specific antibody. SPR sensorgrams of equimolar injections of as-APF over a CM5 chip surface before (**a**), and after (**c**) injection of anti-CKAP4 (epitope region: 552–602) antibody (**b**). CKAP4_525–602_ was immobilized on Fc2 channel by amine-coupling. Fc1 channel was similarly treated but without CKAP4, as the reference

To confirm that the binding of APF to CKAP4 is specific, an antibody competition experiment was performed. The CKAP4_525–602_ and as-APF interaction was selected for the competition assay, because other epitope specific antibodies did not display appropriate reactivity toward their corresponding ligands under our SPR assay conditions. APF binding was measured once under normal conditions and then again after the immobilized CKAP4 ligand was blocked using a specific antibody. The result shown in Fig. 7 clearly indicates that the binding of as-APF to CKAP4_525–602_ was completely abrogated by the CKAP4 antibody (against Aa 552–602 epitope), confirming that the binding between as-APF and CKAP4_525–602_ is specific within this experimental context. The binding specificity between CKAP4_127–360_ and CKAP4_361–524_ with APF could not be determined using this method due to limitations of antibody reactivity under our SPR conditions.

### CKAP4-APF binding can be detected in urine

**Fig. 8:**
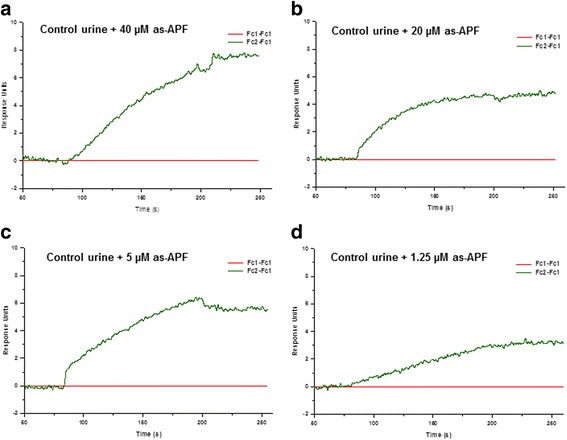
Detection of as-APF in spiked urine. Control urine was mixed 1:1 with as-APF stock (200 μM) solution. Various dilutions of this mixture in SPR running buffer were prepared and injected over a CM5 chip surface immobilized with CKAP4_127–360_ by amine-coupling on the Fc2 channel. Fc1 channel was similarly treated but without CKAP4, as the reference**.** Final concentrations of as-APF were 40 μM (**a**), 20 μM (**b**), 5 μM (**c**), and 1.25 μM (**d**). Sensorgrams were corrected for background interference from urinary constituents by subtracting the RU traces resulting from unspiked urine injections from the RU traces resulting from the corresponding urine dilutions with spiked as-APF, and re-plotted after subtracting the RU trace from the reference (Fc1) channel

In order to determine whether this SPR-based assay employing CKAP4 as a biosensor could be used to detect APF in urine, we used urine from healthy donors spiked with as-APF as a mimetic for IC patient urine. CKAP4_127–360_ was employed as the biosensing ligand, since it bound as-APF with the highest affinity. Various dilutions of the as-APF spiked urine sample were prepared and injected over a CM5 sensor chip surface containing immobilized CKAP4_127–360_ ligand (Fc2 channel). To subtract the background interference from urinary constituents, corresponding dilutions of unspiked urine were also run to serve as individual controls. The representative sensorgrams (Fig. 8a–d) showed that it was possible to detect low concentrations of as-APF in urine using a CKAP4 biosensor in an SPR-based assay. as-APF concentrations as low as 1.25 μM were easily detectable (Fig. 8d). Additional experiments are needed to accurately establish detection limits for this assay. Nevertheless, with further refinements, we expect that it will be possible to detect submicromolar concentrations of urinary as-APF/APF.

## Discussion

This study is the first to report and provide evidence of the direct binding of APF to CKAP4, further confirming that CKAP4 is a physiological receptor for APF. Using SPR and a bead-based approach combined with MS, the reported results demonstrate that the binding sites of APF are located within the extracellular domain of CKAP4. Since CKAP4 is a transmembrane protein, mimicking its biological conformation in vitro is quite challenging. However, its extracellular domain (ED) has unique structural features, including coiled-coil domains that promote self-organization, which enabled the creation of deletion mutants encompassing this region used to assess APF/as-APF binding by SPR.

In our initial attempt to characterize the CKAP4-APF interaction we used full-length CKAP4 and a recombinant variant with the extracellular domain region intact (CKAP4_FL_ and CKAP4_106–602_). Immobilization of these proteins via a His-tag onto the NTA sensor chip surface did not generate sufficient binding with APF; however, when immobilized onto a CM5 chip by amine-coupling, APF binding was detected. These results suggested that immobilization via the His-tag on the N- or C-terminus of the recombinant proteins produced conformational constraints for APF binding and may have fixed their orientation on the sensor chip surface, thus limiting the opportunity to achieve proper conformational folding. In contrast, amine-coupling provided multiple, random chances for the proteins to react with the sensor chip surface through N-terminal and/or internal amine groups (from Lys and Arg), yielding specific orientation(s) that folded correctly, leading to the formation of an active CKAP4 conformation.

CKAP4’s large extracellular domain consists of 474 amino acids that fold into an extensive coiled-coil containing five heptad repeats (residues 468–503) that is predicted to be a leucine zipper (http://www.predictprotein.org/). The sequence of residues that follows (503–602) is predicted to be helical as well (www.ebi.ac.uk/Tools/InterProScan/), and is relatively rich in positively charged, basic amino acids. Examination of this sequence in helical wheel programs (e.g., www.tcdb.org/progs/?tool=pepwheel) shows that the helix is amphipathic, suggesting that this region may also be involved in intermolecular interactions. These structural domains (Fig. 3) guided our strategy to generate deletion mutants of this region in order to define more specific binding site(s) for APF. All of the CKAP4 ED deletion mutants examined in this study exhibited specific binding to active APF. The fitted data from different binding models suggest that distinct regions of the extracellular domain of CKAP4 contribute differentially to APF binding in terms of conformational changes and binding sites. The CKAP4_525–602_ mutant displayed less binding activity to as-APF (100–200 fold less than CKAP4_127–360_ and CKAP4_361–524_), perhaps due to its inability to form a stable binding site after the loss of upstream sequence. Nevertheless, the data suggests that the most C-terminal region of CKAP4 also contributes to overall APF binding. In contrast, the CKAP4_127–360_ and CKAP4_361–524_ mutants displayed robust binding with similar binding kinetics, implying that the core APF binding site(s) are located within the 127 to 524 amino acid region. Indeed, the differential analysis of the data shown in Fig. 5 suggests that there is a high likelihood for CKAP4 to have more than one binding site acting in a coordinated manner to bind APF. Having a clearer picture about the structure of the CKAP4 extracellular domain will help to solve the details of this binding. Additional studies to determine whether there is a minimal domain within this region that generates stronger binding to APF are currently underway; moreover, potential molecular interactions between CKAP4 and two other reported ligands, SP-A and tPA, are being explored.

Another unique aspect of our findings was that as-APF, the desialylated APF analogue, showed greater activity (receptor affinity as well as biological potency) than APF in all our assays, across various technology platforms. APF is a nonapeptide containing a 2,3-sialylated core 1 α-O-linked disaccharide linked to the N-terminal threonine residue (Neu5Acα2 **−** 3Galβ1 **−** 3GalNAcα **−** O **−** TVPAAVVVA) (2). We also surmised that the APF saccharide moiety is required for CKAP4 binding (Fig. 1). Our preliminary studies on the SPR platform revealed that all of the CKAP4 mutants that displayed positive binding response with as-APF and/or APF, showed no binding response with the non-glycosylated APF peptide (Fig. 1). Earlier APF studies, supported in part by activity assays presented here (Fig. 2), have shown that the APF saccharide moiety is an absolute requirement for biological activity, and that such activity does not depend on the presence of the terminal sialic acid [2]. Thus, it is important to note that such structural constraints required for APF’s biological activity are also required for its interaction with CKAP4, which provides further implication of CKAP4’s role as a cellular receptor for APF.

Indications that as-APF has greater binding affinity than APF for CKAP4 and that most structural and biochemical studies to date have focused solely on as-APF give credence to an earlier suggestion that APF may be a precursor of as-APF, perhaps resulting by the action of an intracellular neuraminidase [22, 23]. A recently published structural study has revealed the active APF conformation using as-APF [24]. It has shown that hydrophobic folding within the nine amino acids (TVPAAVVVA), further stabilized by interaction with the saccharide moiety, provides a collapsed conformation for optimal biological function. Thus, addition of the terminal sialic acid moiety can destabilize the hydrophobic core or disturb the conformation to linear form direction, thereby resulting in lower molecular binding activity to CKAP4. Whether or not as-APF is a naturally occurring, active form of APF in pathological conditions such as IC requires further verification. Based on observations that the APF peptide portion is extremely hydrophobic and that the sugar moiety protrudes from the N-terminus, structure-activity relationship studies with APF variants have suggested that the APF peptide may interact with the membrane and thereby present the sugar portion of the molecule for recognition, possibly by a receptor protein [23, 25]. This hypothesis was dependent on whether the APF sugar moiety was important for binding to a putative receptor. Interestingly, our reported results characterizing the CKAP4–APF interaction are in agreement with this hypothesis and indicate a strong requirement of the sugar moiety for binding to CKAP4 (Figs. 1 and 5).

Finally, the results from this study are also significant in terms of their potential clinical application. As mentioned previously, APF has been shown to cause abnormalities in normal bladder epithelial cells that mimic changes seen in explanted cells from IC patients; moreover, its specificity for urine samples and cells from these patients make it a promising urinary biomarker for IC [26–28]. However, validation of APF’s utility as a diagnostic biomarker has been hindered by the absence of specific, sensitive assays to measure its concentration in patient urine. Since we were able to obtain robust and specific APF binding to CKAP4 by SPR, we sought to determine the suitability of CKAP4_127–360_ to quantitate APF in urine. Our results show that immobilized CKAP4_127–360_ can detect APF charged in normal urine in a dose-dependent manner. Thus, with further refinement to enhance sensitivity, this label-free technique employing CKAP4 as a biosensor to detect and measure APF in urine may be a suitable approach for IC diagnosis.

## Conclusions

It has been well established that CKAP4 expression is required for mediating APF’s antiproliferative activity in vitro; however, direct binding of APF to CKAP4 has not been demonstrated previously. In this study, SPR revealed specific binding of APF/as-APF to the CKAP4 extracellular domain (Aa 127–524). APF and as-APF bound CKAP4 specifically but with altered sensitivity. We determined that the CKAP4_127–360_ and CKAP4_361–524_ mutants exhibit improved binding activity to APF as compared to the full-length extracellular domain, making it possible to detect low concentrations of as-APF in urine, thereby establishing a foundation for a non-invasive diagnostic assay for IC. Further, these data have revealed novel APF binding site(s) suggesting that targeting this region of CKAP4 to inhibit APF binding may be a useful strategy for treating IC-related bladder pathology.

## Abbreviations

APF: Antiproliferative factor
CKAP4: Cytoskeleton-associated protein 4
DKK1: Dickkopf1
ED: Extracellular domain
EDC/NHS: 1-ethyl-3-(3-dimethylaminopropyl) carbodiimide hydrochloride/N-hydroxysuccinimide
ER: Endoplasmic recticulum
FBS: Fetal bovine serum
HT: His-tag
IC: Interstitial cystitis
IMAC: Immobilized metal affinity chromatography
LC-MS: Liquid chromatography-mass spectrometry
NTA: Nitrilotriacetic acid
PBS: Phosphate-buffered saline
SP-A: Surfactant protein A
SPR: Surface plasmon resonance
TM: Transmembrane
tPA: Tissue plasminogen activator
VSMC: Vascular smooth muscle cells

## References

[1] Keay S, Kleinberg M, Zhang CO, Hise MK, Warren JW (2000). Bladder epithelial cells from patients with interstitial cystitis produce an inhibitor of heparin-binding epidermal growth factor-like growth factor production. J Urol.

[2] Keay SK, Szekely Z, Conrads TP, Veenstra TD, Barchi JJ, Zhang CO, Koch KR, Michejda CJ (2004). An antiproliferative factor from interstitial cystitis patients is a frizzled 8 protein-related sialoglycopeptide. Proc Natl Acad Sci U S A.

[3] Conrads TP, Tocci GM, Hood BL, Zhang CO, Guo L, Koch KR, Michejda CJ, Veenstra TD, Keay SK (2006). Ckap4/p63 Is a receptor for the frizzled-8 protein-related antiproliferative factor from interstitial cystitis patients. J Biol Chem.

[4] Schweizer A, Ericsson M, Bachi T, Griffiths G, Hauri HP (1993). Characterization of a novel 63 kda membrane protein. Implications for the organization of the er-to-golgi pathway. J Cell Sci.

[5] Schweizer A, Rohrer J, Hauri HP, Kornfeld S (1994). Retention of p63 in an er-golgi intermediate compartment depends on the presence of all three of its domains and on its ability to form oligomers. J Cell Biol.

[6] Klopfenstein DR, Kappeler F, Hauri HP (1998). A novel direct interaction of endoplasmic reticulum with microtubules. EMBO J.

[7] Vedrenne C, Hauri HP (2006). Morphogenesis of the endoplasmic reticulum: beyond active membrane expansion. Traffic.

[8] Vedrenne C, Klopfenstein DR, Hauri HP (2005). Phosphorylation controls climp-63-mediated anchoring of the endoplasmic reticulum to microtubules. Mol Biol Cell.

[9] Planey SL, Zacharias DA (2009). Palmitoyl acyltransferases, their substrates, and novel assays to connect them (review). Mol Membr Biol.

[10] Shahjee HM, Koch KR, Guo L, Zhang CO, Keay SK (2010). Antiproliferative factor decreases akt phosphorylation and alters gene expression via ckap4 in t24 bladder carcinoma cells. J Exp Clin Cancer Res.

[11] Matika CA, Wasilewski M, Arnott JA, Planey SL (2012). Antiproliferative factor regulates connective tissue growth factor (ctgf/ccn2) expression in t24 bladder carcinoma cells. Mol Biol Cell.

[12] Razzaq TM, Bass R, Vines DJ, Werner F, Whawell SA, Ellis V (2003). Functional regulation of tissue plasminogen activator on the surface of vascular smooth muscle cells by the type-ii transmembrane protein p63 (ckap4). J Biol Chem.

[13] Gupta N, Manevich Y, Kazi AS, Tao JQ, Fisher AB, Bates SR (2006). Identification and characterization of p63 (ckap4/ergic-63/climp-63), a surfactant protein a binding protein, on type ii pneumocytes. Am J Physiol Lung Cell Mol Physiol.

[14] Bates SR, Kazi AS, Tao JQ, Yu KJ, Gonder DS, Feinstein SI, Fisher AB (2008). Role of p63 (ckap4) in binding of surfactant protein-a to type ii pneumocytes. Am J Physiol Lung Cell Mol Physiol.

[15] Kimura H, Fumoto K, Shojima K, Nojima S, Osugi Y, Tomihara H, Eguchi H, Shintani Y, Endo H, Inoue M, Doki Y (2016). Ckap4 Is a dickkopf1 receptor and is involved in tumor progression. J Clin Invest.

[16] Kikuchi A, Fumoto K, Kimura H. The dickkopf1-ckap4 axis creates a novel signaling pathway and may represent a molecular target for cancer therapy. Br J Pharmacol. 2017; 10.1111/bph.13863 PMC5727324 28514532

[17] Planey SL, Keay SK, Zhang CO, Zacharias DA (2009). Palmitoylation of cytoskeleton associated protein 4 by dhhc2 regulates antiproliferative factor-mediated signaling. Mol Biol Cell.

[18] Zhang J, Planey SL, Ceballos C, Stevens SM, Keay SK, Zacharias DA (2008). Identification of ckap4/p63 as a major substrate of the palmitoyl acyltransferase dhhc2, a putative tumor suppressor, using a novel proteomics method. Mol Cell Proteomics.

[19] Ling J, Liao H, Clark R, Wong MS, Lo DD (2008). Structural constraints for the binding of short peptides to claudin-4 revealed by surface plasmon resonance. J Biol Chem.

[20] Haas J, Roth S, Arnold K, Kiefer F, Schmidt T, Bordoli L, Schwede T: The protein model portal--a comprehensive resource for protein structure and model information. Database (Oxford) 2013;2013:bat031. 10.1093/database/bat031 PMC3889916 23624946

[21] Roy A, Kucukural A, Zhang Y (2010). I-tasser: a unified platform for automated protein structure and function prediction. Nat Protoc.

[22] Barchi JJ, Kaczmarek P (2009). Short and sweet: evolution of a small glycopeptide from a bladder disorder to an anticancer lead. Mol Interv.

[23] Kaczmarek P, Tocci GM, Keay SK, Adams KM, Zhang CO, Koch KR, Grkovic D, Guo L, Michejda CJ, Barchi JJ (2010). Structure-activity studies on antiproliferative factor (apf) glycooctapeptide derivatives. ACS Med Chem Lett.

[24] Mallajosyula SS, Adams KM, Barchi JJ, MacKerell AD (2013). Conformational determinants of the activity of antiproliferative factor glycopeptide. J Chem Inf Model.

[25] Kaczmarek P, Keay SK, Tocci GM, Koch KR, Zhang CO, Barchi JJ, Grkovic D, Guo L, Michejda CJ (2008). Structure-activity relationship studies for the peptide portion of the bladder epithelial cell antiproliferative factor from interstitial cystitis patients. J Med Chem.

[26] Keay S, Zhang CO, Hise MK, Hebel JR, Jacobs SC, Gordon D, Whitmore K, Bodison S, Gordon N, Warren JW (1998). A diagnostic in vitro urine assay for interstitial cystitis. Urology.

[27] Keay S, Zhang CO, Marvel R, Chai T (2001). Antiproliferative factor, heparin-binding epidermal growth factor-like growth factor, and epidermal growth factor: sensitive and specific urine markers for interstitial cystitis. Urology.

[28] Keay S, Zhang CO, Shoenfelt JL, Chai TC (2003). Decreased in vitro proliferation of bladder epithelial cells from patients with interstitial cystitis. Urology.

